# MR Imaging of Pulmonary Nodules: Detection Rate and Accuracy of Size Estimation in Comparison to Computed Tomography

**DOI:** 10.1371/journal.pone.0156272

**Published:** 2016-06-03

**Authors:** Andrzej Cieszanowski, Antonina Lisowska, Marta Dabrowska, Piotr Korczynski, Malgorzata Zukowska, Ireneusz P. Grudzinski, Ryszard Pacho, Olgierd Rowinski, Rafal Krenke

**Affiliations:** 1 2^nd^ Department of Clinical Radiology, Medical University of Warsaw, Central Clinical Hospital, Warsaw, Poland; 2 Maria Skłodowska-Curie Memorial Cancer Center, Institute of Oncology, Warsaw, Poland; 3 Department of Internal Medicine, Pneumonology and Allergology, Medical University of Warsaw, Warsaw, Poland; 4 Department of Toxicology, Faculty of Pharmacy, Medical University of Warsaw, Warsaw, Poland; Le Fe Health Research Institute, SPAIN

## Abstract

**Objective:**

The aims of this study were to assess the sensitivity of various magnetic resonance imaging (MRI) sequences for the diagnosis of pulmonary nodules and to estimate the accuracy of MRI for the measurement of lesion size, as compared to computed tomography (CT).

**Methods:**

Fifty patients with 113 pulmonary nodules diagnosed by CT underwent lung MRI and CT. MRI studies were performed on 1.5T scanner using the following sequences: T2-TSE, T2-SPIR, T2-STIR, T2-HASTE, T1-VIBE, and T1-out-of-phase. CT and MRI data were analyzed independently by two radiologists.

**Results:**

The overall sensitivity of MRI for the detection of pulmonary nodules was 80.5% and according to nodule size: 57.1% for nodules ≤4mm, 75% for nodules >4-6mm, 87.5% for nodules >6-8mm and 100% for nodules >8mm. MRI sequences yielded following sensitivities: 69% (T1-VIBE), 54.9% (T2-SPIR), 48.7% (T2-TSE), 48.7% (T1-out-of-phase), 45.1% (T2-STIR), 25.7% (T2-HASTE), respectively. There was very strong agreement between the maximum diameter of pulmonary nodules measured by CT and MRI (mean difference -0.02 mm; 95% CI –1.6–1.57 mm; Bland-Altman analysis).

**Conclusions:**

MRI yielded high sensitivity for the detection of pulmonary nodules and enabled accurate assessment of their diameter. Therefore it may be considered an alternative to CT for follow-up of some lung lesions. However, due to significant number of false positive diagnoses, it is not ready to replace CT as a tool for lung nodule detection.

## Introduction

The United States National Lung Screening Trial (NLST) with 53,545 participants, suggested that screening of a high-risk population with low-dose computed tomography (CT) is beneficial in terms of up to 20% reduction of mortality related to lung cancer [1]. Nevertheless, the repetitive use of CT in both screening programs and follow-up of detected pulmonary nodules is associated with significant cumulative radiation exposure and may occasionally lead to development of cancer [2–4]. In this context, an alternative technique with similar sensitivity for the detection of lung lesions, but not associated with radiation exposure, would be more advantageous [5–8].

Advances in magnetic resonance imaging (MRI) technology, including high-performance gradient systems, parallel imaging, increased field homogeneity and multichannel coils allowed implementation of this technique for clinical lung imaging [5, 7]. In recent years, MRI has been increasingly used for the evaluation of different lung diseases including bronchopulmonary dysplasia, cystic fibrosis, cardio-pulmonary vascular abnormalities and intrathoracic tumours [7, 9]. The value of MRI in patients with pulmonary nodules, in terms of lesion detection and characterization, was assessed in several studies. The results of some studies were promising, reporting sensitivity ranging from 40% to 93% [10–22]. However, the presented data were often insufficient to precisely assess the efficacy of MRI for the detection of lesions which carry a relatively high risk of malignancy (defined as >8 mm in size according to both, Fleischner Society and the American College of Chest Physicians). Management of such nodules includes probability of malignancy assessment and often more thorough investigation, including FDG PET, dynamic contrast enhanced CT, and/or biopsy [23–28].

If MRI is to be considered as a potential alternative to CT it will have to achieve the sufficient accuracy for nodule detection and assessment of nodule maximum diameter. Consequently, there were two aims of this prospective study: firstly, to assess the sensitivity of MRI, including sensitivities of specific MRI sequences, for the detection of pulmonary nodules and secondly, to estimate the accuracy of MRI measurement of lesion size in comparison with CT.

## Materials and Methods

The study was approved by the Academic Bioethics Committee of Medical University of Warsaw and all patients participating in the study signed an informed consent.

### Patient Population

Between November 2011 and January 2014, 54 consecutive patients were enrolled for this single-center prospective study. Inclusion criteria were as follows: age ≥18 years, the presence of pulmonary nodule confirmed by CT scan, nodule size between 2 mm and 30 mm, willingness and ability to undergo MRI and participate in the study. Exclusion criteria were contraindications to MR imaging such as pacemakers, metal implants and severe claustrophobia; age <18 years. According to the study protocol, the time interval between CT scan and MRI was not longer than 14 days.

Ultimately, 4 patients were excluded from analysis due to lack of sufficient clinical data or reference imaging studies to validate abnormalities detected during MR imaging. Thus, the study group finally comprised 50 patients (21 male, 29 female) with a mean age of 66.6 years (age range: 18–85 years).

### Computed Tomography

CT scans were obtained using a 16-row and 64-row CT scanners (Light Speed and Optima CT660, respectively; General Electric, Milwaukee, WI, USA). The following scan parameters were applied: 1.25 mm collimation, 0.938–0.984 mm table feed per rotation, 150–300 miliampere second, 120 KV tube voltage. Image reconstructions were performed in axial and coronal orientations using 3 mm slice thickness.

### Magnetic Resonance Imaging

All MRI studies were performed at a 1.5-T unit (Magnetom Avanto, Siemens Medical Solutions, Erlangen, Germany) using explorer gradients (maximum gradient of 40, 40, 45 mT/m along x, y and z axis respectively and slew rate of 200mT/m/ms) and phased-array multicoil system (12 elements).

The images were obtained using broad spectrum of non-contrast enhanced MRI sequences commonly applied for imaging of lung lesions: breath-hold T2-weighted TSE sequence (T2 TSE), T2-weighted TSE sequence with fat-saturation (T2 SPIR), T2-weighted short-tau inversion recovery sequence (T2 STIR), 2D half-Fourier acquisition single-shot turbo spin-echo (T2 HASTE), T1-weighted three-dimensional gradient-echo volumetric interpolated breath-hold examination (T1 VIBE) and T1-weighted out-of-phase images (T1 out-of-phase). Selected parameters of the applied sequences are shown in Table 1.

**Table 1:** Parameters of the applied MRI sequences.

Parameter	T1 VIBE	T2 TSE	T2 STIR	T1 out of phase	T2 SPIR	T2 HASTE
**TR (ms)**	3,02	2500	2800	4,74	2500	800
**TE/TEs (ms)**	1,13	123	96	2,39	123	27
**Flip (st)**	10	150	150	10	150	160
**Turbo factor**	-	51	25	-	51	113
**SENSE factor**	2	2	2	2	2	0
**Plane**	Axial	Axial	Axial	Axial	Axial	Axial
**NSA**	1	1	1	1	1	1
**FOV (mm)**	360	360	400	360	360	360
**RecFOV (%)**	75	100	75	75	100	75
**Matrix**	156x288	256x256	144x256	147x288	256x256	113x256
**Slice thickness**	3	5	5	3	5	5
**Breath-hold**	Yes	Yes	Yes	Yes	Yes	Yes
**Acquisition time (s)**	16''	2'15''	1'52''	20''	2'15''	47''

### Validation of MRI Findings

In 30 patients the benign nature of the nodule was determined based on complete nodule resolution, decreased nodule size or stable nodule dimensions in CT scan performed at least 24 months after the initial diagnosis. Six patients underwent nodule excision. In these patients histopathology revealed 2 lung adenocarcinomas, 2 metastatic malignant tumors (malignant melanoma and breast carcinoma) and 2 benign nonspecific inflammatory nodules. In eight patients follow-up had not been completed at the moment of manuscript preparation. None of the nodules identified in these patients showed radiological progression. Six patients were lost to follow-up and the differentiation between malignant and benign nodule nature could not have been done.

### Image Evaluation

Evaluation of CT and MR images as well as measurements of identified lesions were performed at commercial workstations (Leonardo, Siemens Medical Solution, Erlangen, Germany and Centricity Radiology RA1000, GE Healthcare, Milwaukee, WI, USA). Two radiologists with 24 and 18-year experience in body CT imaging analyzed CT data and two other radiologists with 15 and 10-year experience in body MR imaging read MRI datasets. Readers knew that patients were referred for MRI to study lung nodules. Discrepancies between radiologists, in terms of lesion detection and measurement, were resolved by consensus interpretation, separately for CT and MR images. Each MRI sequence was evaluated for the detection of lung lesions and the sensitivity for each sequence was calculated. If disagreement between MRI sequences occurred, the positive result (detected nodule) was chosen and the combined sensitivity of all MRI sequences was calculated. False negative MRI result was defined as the presence of nodule (confirmed by CT) that was not detected on specific MR images (separate analysis for each MRI sequence was carried out). The number of false positive diagnoses was documented for each MRI sequence. The measurements of the nodule maximum diameter on MR were performed on the image which enabled best lesion delineation.

### Statistical Analysis

Statistical analysis was performed using STATISTICA 10.0 (StatSoft, Inc. Tulsa, OK, USA) and MedCalc 9.5.2.0 (MedCalc Software bvba Ostend, Belgium) software packages. Since participants may have had more than one pulmonary nodule, a per-nodule based approach was used for data analysis. All continuous variables are presented as median and interquartile range (IQR), while categorical variables as number (%), as appropriate. Mann-Whitney U test and Chi-squared test were used to compare categorical variables and continuous variables, as appropriate. The Spearman’s rank correlation coefficient was applied to test correlations between nodule dimensions measured by MRI and CT.

The detection rate of pulmonary nodules was calculated for whole MRI dataset and for individual MRI sequences. Separate analyses have been done for all nodules as well as for following subgroups of lesions (based on Fleischner society recommendation): ≤4 mm, >4–6 mm, >6–8 mm, >8 mm. Diagnostic sensitivity of MRI was defined as the proportion of nodules detected by both CT and MRI to all nodules identified in CT scans. As prespecified, pulmonary nodules were seen in all CT scans included in this study—specificity and negative predictive value of MRI could not have been calculated (no true negative CT results). The Bland-Altman analysis was performed to compare the agreement between the results of CT and MRI measurement of maximum nodule diameter.

## Results

### Lesion Detection

A total of 113 pulmonary nodules, measuring from 2 to 28 mm were identified on CT scans of 50 patients. Thirty-six patients had more than 1 lesion (the maximum number of lesions per patient was 9).

**Fig 1:**
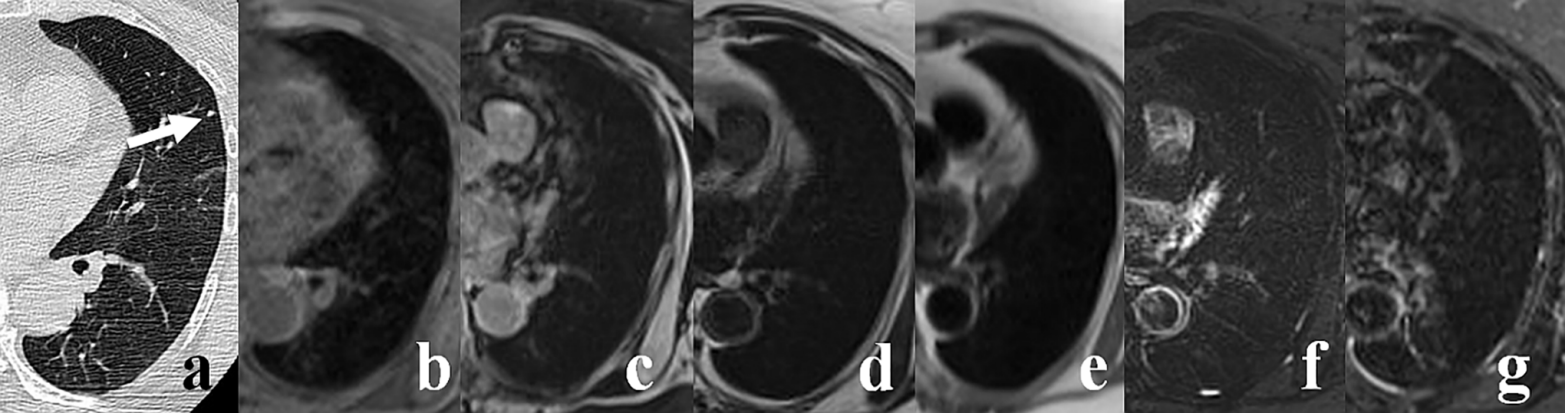
A 77-year-old woman with 2.8 mm pulmonary nodule (arrow) in the left upper lobe (lingula). The nodule is well depicted on MDCT image (A), however it was not identified on corresponding MR images: T1 VIBE (B), T1 out-of-phase (C), T2 TSE (D), T2 HASTE (E), T2 SPIR (F), T2 STIR (G).

**Fig 2:**
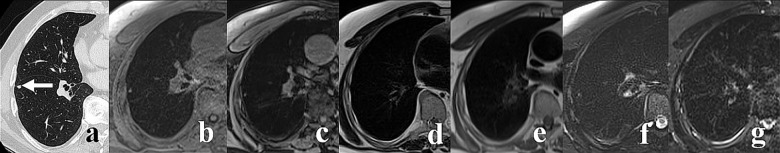
A 62-year-old man with 5.6 mm pulmonary nodule in the right lower lobe (arrow). The lesion is seen on CT (A) as well as on T1 VIBE (B), T1 out-of-phase (C) and T2 SPIR (F) images, whereas T2 TSE (D), T2 HASTE (E) and T2 STIR (G) images were negative.

**Fig 3:**
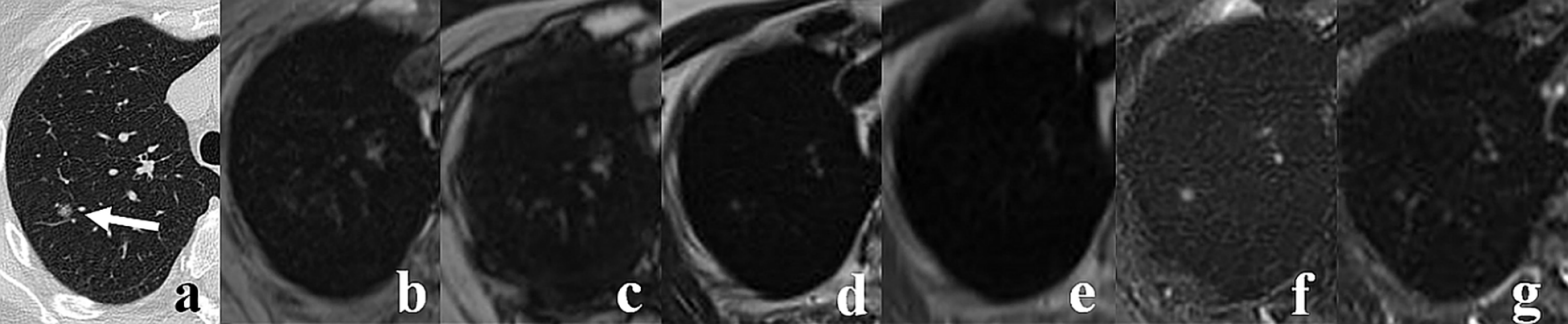
A 71-year-old woman with 7 mm pulmonary nodule in the right upper lobe (arrow). The nodule is demonstrated on CT (A) and on most of T2-weighted sequences, including: T2 TSE (D), T2 SPIR (F) and T2 STIR (G). The readers did not identify this lesion on T1 VIBE (B), T1 out-of-phase (C) and T2 HASTE (E) images.

**Fig 4:**
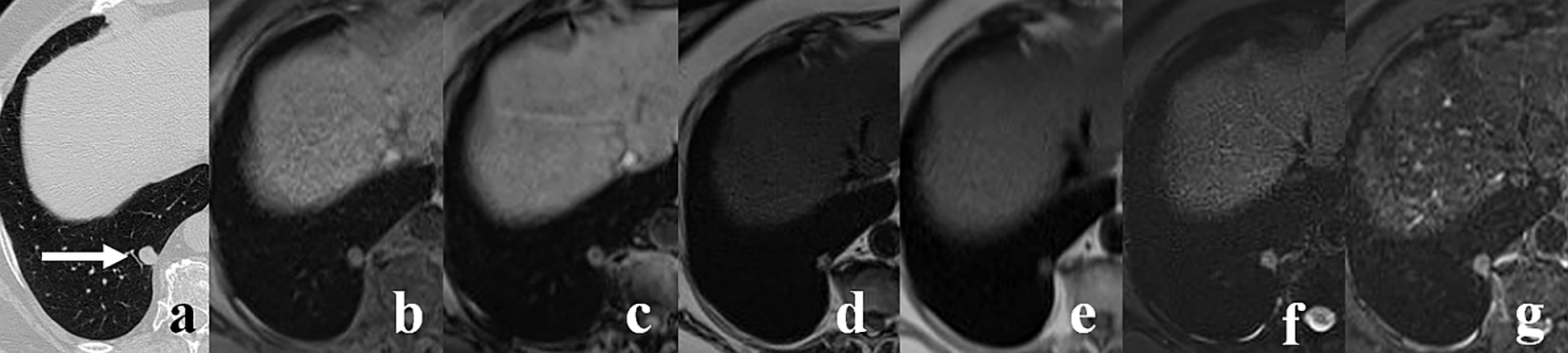
A 71-year-old man with 11.3 mm pulmonary nodule in the right lower lobe (arrow). The nodule is well visualized on CT (A) as well as on all MR images: T1 VIBE (B), T1 out-of-phase (C), T2 TSE (D), T2 HASTE (E), T2 SPIR (F), T2 STIR (G).

MRI correctly depicted 91 pulmonary nodules and failed to identify 22 lesions, yielding the overall sensitivity of 80.5%. All pulmonary nodules larger than 8 mm were identified on MR images (100% sensitivity). The examples of corresponding CT and MRI images of pulmonary nodules of different size are presented in Figs 1–4. The median maximum MRI diameter of lesions detected in CT was 5.5 mm (IQR 5.1–9.0) The median maximum CT diameter of lesions detected in MRI, i.e. 6.9 mm (IQR 5.0–9.0) was significantly larger than that of undetected nodules, i.e. 5.0 mm (IQR 3.0–6.0) (*P* = 0.0001). The maximum diameter of the largest lesion not detected by MRI was 7 mm. Almost one half (10/22) of undetected lesions were ≤4 mm. The size of the remaining MRI undetected nodules was as follows: >4–6 mm (9 nodules) and >6–8 mm (3 nodules). The maximum diameters of all nodules on CT and MR images, are available in S1 Table.

The sensitivity of MRI for the detection of different categories of pulmonary nodules (according to Fleischner society recommendation) is presented in Table 2. Calcifications were depicted on CT scans in 8 of 113 pulmonary nodules. Seven of these nodules (87.5%) were identified on MR images.

**Table 2:** The sensitivity of MRI for the detection of different subgroups of pulmonary nodules (according to the Fleischner Society nodule size category).

Diameter of pulmonary nodules	Number of lesions	Detected by MRI	Sensitivity of MRI
**≤4 mm**	21	12	57.1%
**>4–8 mm**	64	51	79.7%
***>4–6 mm***	*40*	*30*	*75%*
***>6–8 mm***	*24*	*21*	*87*.*5%*
**>8 mm**	28	28	100%
**All lesions**	113	91	80.5%

The T1 VIBE sequence in axial plane showed the highest sensitivity for the detection of pulmonary nodules (69%), followed by T2 SPIR, T2 TSE in axial plane along with T1 out-of-phase, T2 STIR and T2 HASTE (Figs 1–4). Furthermore, T1 VIBE sequence enabled visualization of all lung lesions >8 mm. The sensitivities and false positive diagnoses of all applied MRI sequences for the detection of lung lesions are displayed in Table 3. Lung lesions falsely reported on MR images (false positive diagnoses, Table 3) had average maximum diameter of 4.4 mm; all were smaller than 10 mm.

**Table 3:** The sensitivity for the detection of pulmonary nodules and false positive diagnoses of applied MRI sequences.

MRI Sequence	Sensitivity (all lesions)	Sensitivity(lesions >4–8 mm)	Sensitivity (lesions >8mm)	Number of false positive diagnoses
**T2 TSE (axial plane)**	48.7%	40.6%	85.7%	24
**T2 SPIR (axial plane)**	54.9%	51.6%	82.1%	10
**T2 STIR (axial plane)**	45.1%	35.9%	78.6%	48
**T2 HASTE (axial plane)**	25.7%	20.3%	50%	1
**T1 out-of-phase (axial plane)**	48.7%	37.5%	92.9%	5
**T1 VIBE (axial plane)**	69%	64.1%	100%	14

### Analysis of Lesion Size

**Fig 5:**
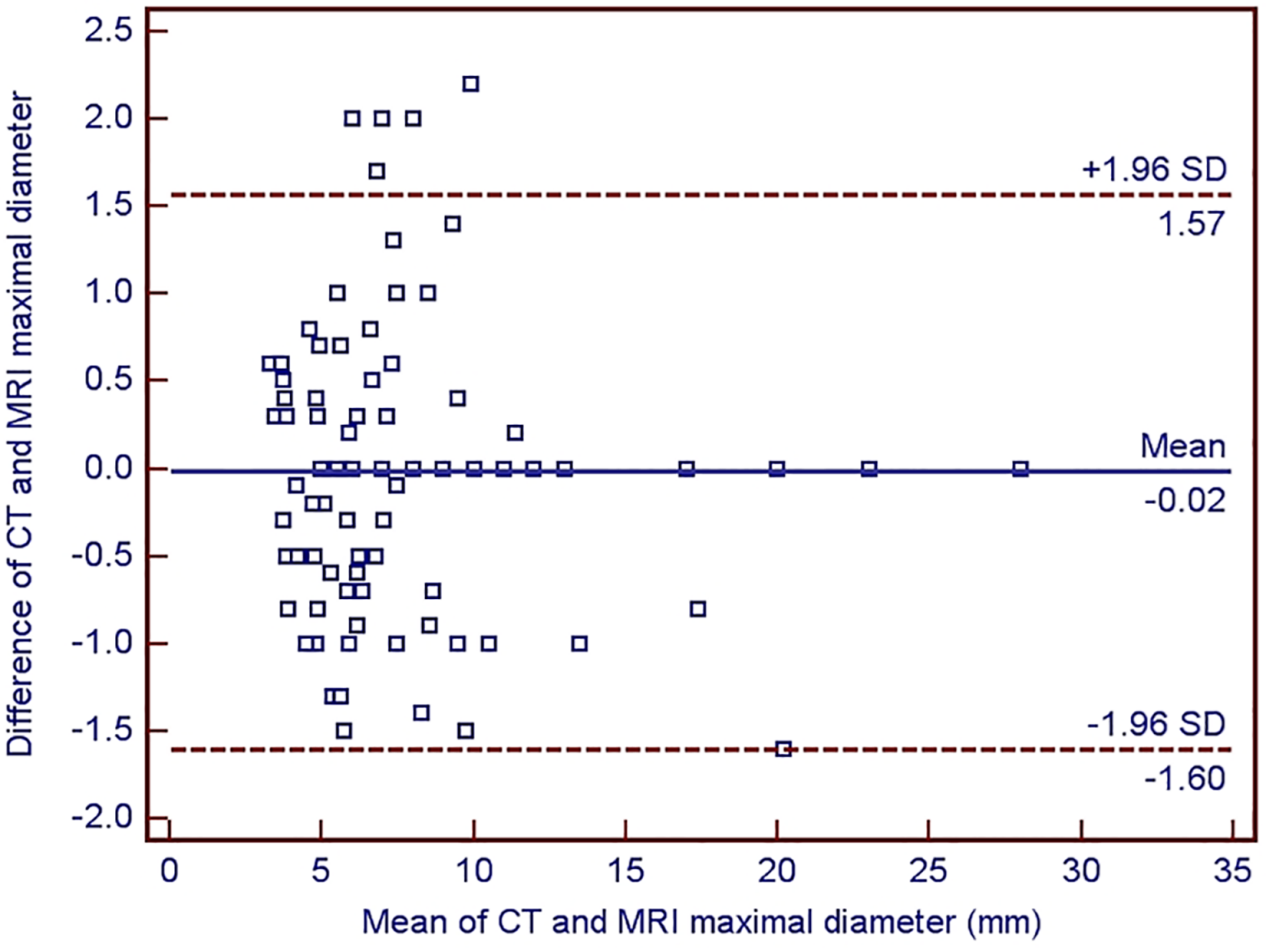
The Bland-Altman plot showing the agreement between the maximum diameter of 91 pulmonary nodules measured by both CT and MRI.

According to CT measurements, the average maximum diameter of the nodules was 7.1 mm. There were 91 nodules detected in both CT scans and MR images. There was a very strong positive correlation between the maximum diameter of pulmonary nodules measured by CT and MRI (*r* = 0.95; P<0.0000). The Bland-Altman plot demonstrated a very strong agreement between maximal nodule diameter measured in CT and MRI (Fig 5).

## Discussion

Pulmonary nodules are increasingly found on chest CT scans. Since only a small proportion of these nodules are malignant, CT follow-up is recommended to demonstrate no growth which is believed to be a strong evidence of benign etiology. However, some authors raise the issue of increased cancer risk related to cumulative radiation dose [2–4]. In this context, the use of MR imaging may be considered as an attractive alternative to CT scan. As the probability of malignancy is closely related to the size of the nodule [29, 30], it is important to evaluate the accuracy of MRI in detection of pulmonary nodules in relation to the nodule size. In our study the sensitivity of MRI for the detection of pulmonary nodules >4–6 mm and >6–8 mm was satisfactory (75 and 87.5%, respectively) and for lesions >8 mm it reached 100%. It must be admitted, however, that we noted significant number of false positive diagnoses generated by MRI. They were more frequent on T2-weighted turbo spin-echo images (T2 STIR– 48, T2 TSE– 34), than on T1-weighted gradient-recalled echo images (T1 VIBE– 14, T1 out-of-phase-5). On the other hand, the average diameter of falsely diagnosed lung lesion was small (4.4 mm) and none of these nodules was larger than 10 mm.It should be emphasized that false negative and false positive results have also been reported in CT imaging. The percentage of these results largely depends on the aim of the study and applied definition of false negative and false positive findings. In studies aimed to detect all malignant pulmonary nodules, a false positive finding is usually defined as a nonmalignant nodule that was misclassified as probably malignant and referred for surgical resection [31]. When the aim of the study is to detect all pulmonary nodules, the percentage of false negative and false positive finding depends on several factors, including scanning parameters and the method of CT analysis (radiologist or computer aided detection) [32]. Nonetheless, due to a significant number of false positive diagnoses produced by MRI, we assume that this technique is still not ready to replace CT as a tool for lung nodule detection. Furthermore, MRI is less available and more expensive than CT. Thus, considering high overall sensitivity of MRI for the detection of significant lung nodules and lack of ionizing radiation, we believe that this method may be an interesting alternative for follow-up of pulmonary nodules detected by CT.

Our study showed that T1 VIBE sequence accomplished the highest sensitivity (69%) for lung lesion detection (Figs 2 and 4). The similar results were reported by Heye et al. and Yi et al. [11, 15], whereas other authors recommended other techniques, including T2 HASTE [12, 13, 16], T2 STIR [14] or Dixon-based MR imaging [21]. These discrepancies may be attributed to different hardware and software implemented in these studies. Some differences in patients and nodule characteristics could also have an impact on the final results. Authors who reported high detection rates of T2 HASTE and T2 STIR sequences [12–14, 16, 19] studied patients with significant number of malignant, often metastatic, lesions which are usually well depicted on T2-weighted images as hyperintense nodules embedded in the signal void of aerated lungs. The results of our study indicate that the T1 VIBE could be the optimal technique for the detection of lung lesions. This is consistent with the reports of Heye et al., and Both et al., who found better performance of a T1 VIBE sequence as compared to a T2 HASTE sequence [15, 33]. Twenty-three of 92 lesions larger than 4 mm were not seen on T1 VIBE images (Fig 3). Since 7 of these nodules were depicted on T2-weighted images (Fig 3), the sensitivity of three combined sequences (T1 VIBE, T2 TSE, T2 SPIR) for the detection of lesions >4 mm increased from 75% (for T1 VIBE sequence only) to 82.6%. Consequently, we recommend inclusion of T2-weighted turbo spin-echo sequence into MRI lung imaging protocol.

It has previously been suggested that T1 VIBE sequence enables better visualization of calcified, typically benign nodules which may demonstrate moderate or even high signal intensity on T1-weighted images, while on T2-weighted images they usually have low signal intensity and often are not visible [11, 13]. Our study did not confirm this observation, though the number of analyzed lesions containing calcifications was small.

Besides the evaluation of MRI as the method of pulmonary nodule detection, our study aimed at assessment of the accuracy of MRI based measurement of maximal nodule diameter. If MRI is to be considered as an alternative method for follow-up of pulmonary nodules, it has to demonstrate a high accuracy in lesion size measurement. In one earlier study addressing this issue, Heye et al. found a strong correlation between maximal nodule diameter measured by MRI and CT [15]. A similar, very strong correlation between measurements by both imaging modalities was demonstrated in our study. More importantly, Bland-Altman analysis showed very good agreement between the size of nodule measured in CT vs. in MRI. In 95% of nodules the difference between the diameter measured in CI vs. in MRI ranged between -1.6 and 1.57 mm (Fig 5).

Some limitations of our study should be mentioned. We did not apply dedicated software with automated or semi-automated three-dimensional volume analysis. According to the study protocol, only patients with pulmonary nodules detected by chest CT scan were examined with MRI. Thus, there were no patients with “true negative” findings in our study group. Therefore the specificity and the negative predictive value of MRI for nodule detection could not have been calculated. Definite histopathological diagnosis was available in 6 patients only. Albeit the proportion of malignant nodules was small (8.7%), it was still somewhat higher than that found in other studies [20, 26]. The study group was limited to 50 patients and our results, even though encouraging, require further confirmation in larger populations. Furthermore, contrast-enhanced sequences were not executed, although we deliberately aimed to evaluate the accuracy of a simple, non contrast-enhanced protocol of lung MRI.

Although the differentiation between malignant and benign nodule based on MRI was beyond the scope of our study, we realize limitations associated with the use of MRI. As compared to CT scan, MRI shows inferior spatial and temporal resolutions, and therefore preclude confident assessment of some morphological characteristics of nodules that correlate with likelihood of malignancy [26–27]. Moreover, susceptibility artifacts from the lungs and artifacts related to patient motion as well as noise from lung parenchyma may lead to a significant number of false positive diagnoses of pulmonary nodules (Table 3). On the other hand, MRI is a radiation-free technique, which proved to be an effective method of reliable evaluation of lesion size. Hence, it could be implemented for the assessment of its doubling time, facilitating distinction between benign and malignant nodules.

## Conclusions

Our study results confirmed that current MRI techniques have a high sensitivity for the detection of pulmonary nodules. Comparison of a wide range of MRI sequences previously applied for lung imaging showed that a T1 VIBE sequence had the highest sensitivity, which further increased when combined with T2 TSE sequences. Similarly to a recent study [15] we demonstrated that MRI can accurately determine lung nodule size. Therefore, we believe that due to lack of ionizing radiation MRI may be an interesting alternative for follow-up of pulmonary nodules. However, due to significant number of false positive diagnoses, it is not ready to replace CT as a tool for lung nodule detection.

## Supporting Information

S1 Table Maximum diameters of 113 pulmonary nodules in 50 patients on MR and CT images. (DOCX)

## References

[1] National Lung Screening Trial Research Team, Aberle DR, Adams AM, Berg CD, Black WC, Clapp JD, Fagerstorm RM et al Reduced lung-cancer mortality with low-dose computed tomographic screening. N Engl J Med. 2011; 365(5):395–409. 10.1056/NEJMoa1102873 21714641 PMC4356534

[2] Bach PB, Mirkin JN, Oliver TK, Azzoli CG, Berry DA, Brawley OW et al Benefits and harms of CT screening for lung cancer: a systematic review. JAMA. 2012; 307(22):2418–2429. 10.1001/jama.2012.5521 22610500 PMC3709596

[3] Sodickson A, Baeyens PF, Andriole KP, Prevedello LM, Nawfel RD, Hanson R et al, Recurrent CT, cumulative radiation exposure, and associated radiation-induced cancer risks from CT of adults. Radiology. 2009; 251(1):175–184. 10.1148/radiol.2511081296 19332852

[4] Brenner DJ. Radiation risks potentially associated with low-dose CT screening of adult smokers for lung cancer. Radiology; 2004; 231(2):440–445. 15128988 10.1148/radiol.2312030880

[5] Sieren JC, Ohno Y, Koyama H, Sugimura K, McLennan G. Recent technological and application developments in computed tomography and magnetic resonance imaging for improved pulmonary nodule detection and lung cancer staging. J Magn Reson Imaging. 2010; 32(6):1353–1369. 10.1002/jmri.22383 21105140 PMC3058763

[6] Biederer J, Hintze C, Fabel M. MRI of pulmonary nodules technique and diagnostic value. Cancer Imaging. 2008; 8:125–130. 10.1102/1470-7330.2008.0018 18519226 PMC2413430

[7] Wielpütz M, Kauczor HU. MRI of the lung: state of the art. Diagn Interv Radiol. 2012; 18(4):344–53. 10.4261/1305-3825.DIR.5365-11.0 22434450

[8] Biederer J, Mirsadraee S, Beer M, Molinari F, Hintze C, Bauman G et al MRI of the lung (3/3)—current applications and future perspectives. Insights Imaging. 2012; 3(4):373–386. 10.1007/s13244-011-0142-z 22695943 PMC3481076

[9] Biederer J, Beer M, Hirsch W, Wild J, Fabel M, Puderbach M, et al MRI of the lung (2/3) Why … when … how? Insights Imaging. 2012; 3(4):355–371. 10.1007/s13244-011-0146-8 22695944 PMC3481084

[10] Kersjes W, Mayer E, Buchenroth M, Schunk K, Fouda N, Cagil H. Diagnosis of pulmonary metastases with turbo-SE MR imaging. Eur Radiol. 1997; 7(8):1190–1194. 9377498 10.1007/s003300050272

[11] Yi CA, Jeon TY, Lee KS, Lee JH, Seo JB, Kim YK et al 3T MRI: usefulness for evaluating primary lung cancer and small nodules in lobes not containing primary tumors. AJR Am J Roentgenol. 2007; 189(2):386–392. 17646465 10.2214/AJR.07.2082

[12] Schroeder T, Ruehm SG, Debatin JF, Ladd ME, Barkhausen J, Goehde SC. Detection of pulmonary nodules using a 2D HASTE MR sequence: comparison with MDCT. AJR Am J Roentgenol. 2005; 185(4):979–984. 16177419 10.2214/AJR.04.0814

[13] Vogt FM, Herborn CU, Hunold P, Lauenstein TC, Schröder T, Debatin JF et al HASTE MRI versus chest radiography in the detection of pulmonary nodules: comparison with MDCT. AJR Am J Roentgenol. 2004; 183(1):71–78. 15208113 10.2214/ajr.183.1.1830071

[14] Luboldt W, Wetter A, Eichler K, Vogl TJ, Wagner TO, Seemann MD. Determination of the optimal MRI sequence for the detection of malignant lung nodules. Eur J Med Res. 2006; 11(8):336–342. 17052969

[15] Heye T, Ley S, Heussel CP, Dienemann H, Kauczor HU, Hosch W et al Detection and size of pulmonary lesions: how accurate is MRI? A prospective comparison of CT and MRI. Acta Radiol. 2012; 53(2):153–160. 10.1258/ar.2011.110445 22287146

[16] Sommer G, Tremper J, Koenigkam-Santos M, Delorme S, Becker N, Biederer J et al Lung nodule detection in a high- risk population: comparison of magnetic resonance imaging and low-dosecomputed tomography. Eur J Radiol. 2014; 83(3):600–605. 10.1016/j.ejrad.2013.11.012 24364923

[17] Baumann T, Ludwig U, Pache G, Gall C, Saueressig U, Fisch D et al Detection of pulmonary nodules with move-during-scan magnetic resonance imaging using a free-breathing turbo inversion recovery magnitude sequence. Invest Radiol. 2008; 43(6):359–367. 10.1097/RLI.0b013e31816901fa 18496040

[18] Biederer J, Schoene A, Freitag S, Reuter M, Heller M. Simulated pulmonary nodules implanted in a dedicated porcine chest phantom: sensitivity of MR imaging for detection. Radiology. 2003; 227(2):475–483. 12649421 10.1148/radiol.2272020635

[19] Bruegel M, Gaa J, Woertler K, Ganter C, Waldt S, Hillerer C et al MRI of the lung: value of different turbo spin-echo, single-shot turbo spin-echo, and 3D gradient-echo pulse sequences for the detection of pulmonary metastases. J Magn Reson Imaging. 2007; 25(1):73–81. 17154370 10.1002/jmri.20824

[20] Wu NY, Cheng HC, Ko JS, Cheng YC, Lin PW, Lin WC et al Magnetic resonance imaging for lung cancer detection: experience in a population of more than 10,000 healthy individuals. BMC Cancer. 2011; 11:242 10.1186/1471-2407-11-242 21668954 PMC3136423

[21] Stolzmann P, Veit-Haibach P, Chuck N, Rossi C, Frauenfelder T, Alkadhi H et al Detection rate, location, and size of pulmonary nodules in trimodality PET/CT-MR: comparison of low-dose CT and Dixon-based MR imaging. Invest Radiol. 2013; 48(5):241–246. 10.1097/RLI.0b013e31826f2de9 23070096

[22] Regier M, Schwarz D, Henes FO, Groth M, Kooijman H, Begemann PG et al Diffusion-weighted MR-imaging for the detection of pulmonary nodules at 1,5Tesla: Intraindividual comparison with multidetector computed tomography. J Med Imaging Radiat Oncol. 2011; 55(3):266–274. 10.1111/j.1754-9485.2011.02263.x 21696559

[23] MacMahon H, Austin JH, Gamsu G, Herold CJ, Jett JR, Naidich DP et al Guidelines for management of small pulmonary nodules detected on CT scans: a statement from the Fleischner Society. Radiology. 2005; 237(2):395–400. 16244247 10.1148/radiol.2372041887

[24] Albert RH, Russell JJ. Evaluation of the solitary pulmonary nodule. Am Fam Physicians. 2009; 80(8):827–831. 19835344

[25] Khan AN, Al-Jahdali HH, Irion KL, Arabi M, Koteyar SS. Solitary pulmonary nodule: A diagnostic algorithm in the light of current imaging technique. Avicenna J Med. 2011; 1(2):39–51. 10.4103/2231-0770.90915 23210008 PMC3507065

[26] McWilliams A, Mayo J. Computed tomography-detected noncalcified pulmonary nodules. A review of evidence for significance and management. Proc Am Thorac Soc. 2008; 5(9):900–904. 10.1513/pats.200809-111QC 19056713

[27] Choromańska A, Macura KJ. Evaluation of solitary pulmonary nodule detected during computed tomography examination. Pol J Radiol. 2012; 77(2):22–34. 22844306 10.12659/pjr.882967 PMC3403798

[28] Gould MK, Donington J, Lynch WR, Mazzone PJ, Midthun DE, Naidich DP et al Evaluation of individuals with pulmonary nodules: when is it lung cancer? Chest. 2013; 143(5 Suppl):93S–120S. 10.1378/chest.12-2351 PMC3749714 23649456

[29] McWilliams AM, Mayo JR, Ahn MI, MacDonald SL, Lam SC. Lung cancer screening using multi-slice thin-section computed tomography and autofluorescence bronchoscopy. J Thorac Oncol. 2006; 1(1):61–68. 17409828

[30] Swensen SJ, Jett JR, Hartman TE. CT screening for lung cancer: five-year prospective experience. Radiology. 2005; 235(1):259–265. 15695622 10.1148/radiol.2351041662

[31] Henschke CI, Yip R, Yankelevitz DF, Smith JP. Definition of a positive test result in computed tomography screening for lung cancer: a cohort study. Ann Intern Med. 2013; 158(4): 246–252. 10.7326/0003-4819-158-4-201302190-00004 23420233

[32] Rubin GD, Lyo JK, Paik DS, Sherbondy AJ, Chow LC, Leung AN et al Pulmonary nodules on multi-detector row CT scans: performance comparison of radiologists and computer aided detection. Radiology. 2005; 234(1):274–283. 15537839 10.1148/radiol.2341040589

[33] Both M, Schultze J, Reuter M, Bewig B, Hubner R, Bobis I et al Fast T1- and T2-weighted pulmonary MR-imaging in patients with bronchial carcinoma. Eur J Radiol. 2005; 53(3):478–488. 15741023 10.1016/j.ejrad.2004.05.007

