# Supplementary material for: MR Imaging of Pulmonary Nodules: Detection Rate and Accuracy of Size Estimation in Comparison to Computed Tomography
Source: PLoS One. 2016 Jun 3;11(6):e0156272. doi: 10.1371/journal.pone.0156272 (PMC4892605; doi:10.1371/journal.pone.0156272)
Supplement: S1 Table — (DOCX) [file pone.0156272.s001.docx]

Supplementary Material

Table A. Maximum diameters of 113 pulmonary nodules in 50 patients on MR and CT images.

|  | MRI: Maximum  diameter | CT: Maximum diameter |
| --- | --- | --- |
| 1 | 10 | 9 |
| 2 | 13 | 13 |
| 3 | 6,5 | 5 |
| 4 | 8 | 8 |
| 5 | 3 | 3,6 |
| 6 | 4,6 | 5,3 |
| 7 | 28 | 28 |
| 8 | 6 | 8 |
| 9 | 5 | 5 |
| 10 | 5,3 | 4,3 |
| 11 | 3,5 | 4 |
| 12 | 7 | 9 |
| 13 | 7 | 6,5 |
| 14 | 9,3 | 9,7 |
| 15 | 11 | 11 |
| 16 | 6,4 | 6,9 |
| 17 | 5,6 | 5 |
| 18 | 5,8 | 6 |
| 19 | 6,2 | 7 |
| 20 | 9 | 9 |
| 21 | 20 | 20 |
| 22 | 6 | 6,3 |
| 23 | 8,6 | 10 |
| 24 | 3,9 | 3,6 |
| 25 | 3,3 | 3,6 |
| 26 | 9 | 8,3 |
| 27 | 3,7 | 4 |
| 28 | 3,4 | 4 |
| 29 | 5,6 | 6 |
| 30 | 6,7 | 6 |
| 31 | 4,6 | 5 |
| 32 | 7,5 | 7,4 |
| 33 | 6 | 5,7 |
| 34 | 5,2 | 5 |
| 35 | 6 | 4,7 |
| 36 | 4,1 | 3,6 |
| 37 | 11,3 | 11,5 |
| 38 | 17,8 | 17 |
| 39 | 8,2 | 10 |
| 40 | 5 | 6 |
| 41 | 4,2 | 5 |
| 42 | 7 | 9 |
| 43 | 4,8 | 4,6 |
| 44 | 18,4 | 19,4 |
| 45 | 6,7 | 8 |
| 46 | 10,5 | 9 |
| 47 | 6,4 | 5,4 |
| 48 | 7 | 8,1 |
| 49 | 7 | 7,6 |
| 50 | 4,2 | 4 |
| 51 | 4,7 | 5 |
| 52 | 6,2 | 5,5 |
| 53 | 9 | 7,6 |
| 54 | 9,5 | 11 |
| 55 | 6 | 5,7 |
| 56 | 7 | 7,3 |
| 57 | 6,5 | 5,9 |
| 58 | 17 | 17 |
| 59 | 5,3 | 4,5 |
| 60 | 5,3 | 5,6 |
| 61 | 3,3 | 3,5 |
| 62 | 5 | 7 |
| 63 | 14 | 13 |
| 64 | 10 | 10 |
| 65 | 7 | 7 |
| 66 | 5 | 5 |
| 67 | 4 | 4 |
| 68 | 11 | 10 |
| 69 | 8 | 9 |
| 70 | 7,2 | 6,9 |
| 71 | 8 | 7 |
| 72 | 9 | 9 |
| 73 | 6 | 6 |
| 74 | 5,5 | 5,5 |
| 75 | 10 | 10 |
| 76 | 6,3 | 5 |
| 77 | 6 | 6 |
| 78 | 7 | 7 |
| 79 | 6 | 6 |
| 80 | 7 | 8 |
| 81 | 5 | 4,5 |
| 82 | 23 | 23 |
| 83 | 4,5 | 4 |
| 84 | 6,5 | 6 |
| 85 | 9 | 9 |
| 86 | 12 | 12 |
| 87 | 7 | 7 |
| 88 | 3,6 | 4 |
| 89 | 8 | 8 |
| 90 | 7 | 7 |
| 91 | 6,5 | 7 |
|  |  |  |
| 92 |  | 3 |
| 93 |  | 5 |
| 94 |  | 2,4 |
| 95 |  | 5 |
| 96 |  | 3 |
| 97 |  | 6 |
| 98 |  | 4,0 |
| 99 |  | 3 |
| 100 |  | 3,5 |
| 101 |  | 6 |
| 102 |  | 5,9 |
| 103 |  | 5 |
| 104 |  | 3,8 |
| 105 |  | 7 |
| 106 |  | 6 |
| 107 |  | 5,5 |
| 108 |  | 2,7 |
| 109 |  | 6,6 |
| 110 |  | 2,5 |
| 111 |  | 7 |
| 112 |  | 3 |
| 113 |  | 6 |
